# Diagnosis and management of symptoms associated with vulvovaginal atrophy: expert opinion on behalf of the Italian VVA study group^*^

**DOI:** 10.1080/09513590.2016.1183627

**Published:** 2016-05-17

**Authors:** Rossella E. Nappi, Nicoletta Biglia, Angelo Cagnacci, Costantino Di Carlo, Stefano Luisi, Anna Maria Paoletti

**Affiliations:** ^a^ Research Center for Reproductive Medicine, Gynecological Endocrinology and Menopause, IRCCS S. Matteo Foundation, Department of Clinical, Surgical, Diagnostic and Paediatric Sciences, University of Pavia, Pavia, Italy; ^b^ Obstetrics and Gynecology, University of Turin, School of Medicine Ospedale Mauriziano Umberto I, Turin, Italy; ^c^ Gynecology and Obstetrics, University of Modena and Reggio Emilia, Azienda Policlinico of Modena, Modena, Italy; ^d^ Gynecology and Obstetrics, Department of Neurosciences and Reproductive Sciences, University of Naples Federico II, Naples, Italy; ^e^ Obstetrics and Gynecology, Department of Molecular and Developmental Medicine, University of Siena, Policlinico “Le Scotte”, Siena, Italy; ^f^ Obstetrics and Gynecology, Department of Surgical Sciences, University of Cagliari, AOU Cagliari, Italy

**Keywords:** Diagnosis, expert opinion, management, vulvovaginal atrophy, VVA

## Abstract

Vulvovaginal atrophy (VVA) is a chronic disorder that commonly occurs in postmenopausal women, whose symptoms are recognized among the most frequent and bothersome symptoms associated with menopause. The principal therapeutic goal in managing VVA is to relieve symptoms as well as to restore the vaginal environment to a healthy state. However, despite its high prevalence and negative impact on quality of life, VVA is underreported by women, underrecognized by gynecologists, and therefore, undertreated. In the light of the new development of treatment options for VVA, we here provide an updated expert opinion on the management of VVA. In particular, we strongly recommend that HCPs proactively start an open discussion with their postmenopausal patients about urogenital symptoms. Treatment should be started as early as the first symptoms of VVA occur and should be maintained over time, due to the chronicity of the conditions. Many treatment options are now available and therapy should be individualized, taking the woman’s preference in consideration.

## Chinese abstract

外阴阴道萎缩(VVA)是绝经后妇女常见的一种慢性疾病, 其症状被认为是绝经相关症状中最常见和最麻烦的。VVA管理中主要治疗目标是缓解症状及重建健康的阴道环境。尽管VVA患病率高, 对妇女的生活质量也有负面影响, 但妇女往往不向医生报告, 妇科医师也没有意识到, 因此该问题也没有得到治疗。我们根据VVA治疗方案的新进展, 在这里提供一个关于VVA管理的新的专家意见。我们特别强烈建议保健专业人员要主动和他们的绝经后患者展开关于泌尿生殖道症状的开放性的讨论。治疗应该在刚刚出现VVA症状的时候就开始, 并且鉴于其慢性的特点, 治疗应维持一段时间。很多治疗方法是可以应用的, 但是考虑到妇女的不同情况, 治疗应该个体化。

## Introduction

Vulvovaginal atrophy (VVA) is a chronic disorder that commonly occurs in postmenopausal women. The symptoms are usually progressive in nature and deteriorate with time from the menopausal transition. The most prevalent symptoms are vaginal dryness, vaginal irritation, itching and soreness, dyspareunia, vaginal bleeding associated with intercourse, and atypical discharge [1–4]. VVA is due to loss of estrogen production in postmenopausal women [5]. The term genitourinary syndrome of menopause (GSM) has been proposed following a consensus conference held in May 2013 [6]. GSM includes not only VVA but also signs and symptoms involving urinary organs and the pelvic floor. We here focus on VVA symptoms that, together with vasomotor symptoms, are recognized as the most frequent and bothersome symptoms associated with menopause and can have a large impact on the quality of life (QoL) of the menopausal woman. The principal therapeutic goal in managing VVA is to relieve symptoms as well as to restore the vaginal environment to a healthy state [2].

In recent large cohort surveys in Western populations, 45–63% of postmenopausal women reported that they had experienced vulvovaginal symptoms [7], most commonly vaginal dryness; other commonly reported symptoms include dyspareunia, vaginal irritation, itching sensation, vaginal tenderness, and vaginal bleeding or spotting during intercourse [7–9]. The VIVA (Vaginal Health: Insights, Views & Attitudes) international survey obtained information from 3520 postmenopausal women aged 55–65 years living in Europe and the US, reporting 45% of women experiencing vaginal symptoms [8]. A very recent analysis of the data from the International Women’s Health (IWH) Study, about postmenopausal women aged 40–75 years in the US and major European countries, showed that the prevalence of VVA symptoms varied between 40% [Germany] and 54% [Spain], with half of women reporting their symptoms as either moderate or severe [10]. Moreover, VVA symptoms were associated with clinically meaningful decrements in QoL, comparable to serious conditions such as arthritis, chronic obstructive pulmonary disease, asthma, and irritable bowel syndrome. In Italy, a multicenter observational study, the AGATA study, recruited 913 females, aged 59.3 ± 7.4 years, asking for a routine gynecological examination. Based on patient’s sensation of vaginal dryness, any objective sign of VVA and a vaginal pH > 5, VVA was diagnosed with a prevalence ranging from 64.7% to 84.2%, starting from 1 to 6 years after menopause. Symptoms reported by women with VVA were vaginal dryness (100%), followed by dyspareunia, burning, itching, and dysuria. Only 30% of women had had a previous diagnosis of VVA/GSM.

The CLOSER (The CLarifying Vaginal Atrophy’s Impact On SEx and Relationships) survey evaluated the impact of VVA on the physical and emotional aspects of sexual relationships between 4100 postmenopausal women and their male partners across Europe, Canada, and the US [11]. Having sex less often and less satisfying, and putting off having sex (women: 35%, men: 14%) were the main effects of VVA. The European REVIVE Study [12], on a surveyed sample of 3768 postmenopausal women aged 45–75 years, found that the most common VVA symptom was vaginal dryness (70%), and that VVA had a significant impact on the ability to be intimate, to enjoy sexual intercourse, and to feel sexual spontaneity, with an overall reduction of sexual drive.

Despite its high prevalence and negative impact on QoL, VVA is underreported by women, underrecognized by gynecologists, and therefore, undertreated [13,14]. There are multiple reasons for this [14,15]. Women feel embarrassed to complain about this problem, health-care professionals (HCPs) also feel uncomfortable discussing sexual issues and have limited time to face such a discussion; moreover, they often are not fully aware of the available treatment options that may be both hormonal and nonhormonal. Improved management of VVA symptoms is required to alleviate the impact of VVA on the QoL of affected women. The North American Menopause Society (NAMS) in 2013 and the European Menopause Andropause Society (EMAS) in 2015 focused on VVA and its treatment, underlining that clinicians can improve the sexual health and QoL of postmenopausal women by educating women about, diagnosing, and appropriately managing symptomatic VVA [16–18]. Palacios and Mejias [19] recently reviewed the drugs for the treatment of menopausal symptoms, showing that there are increasing numbers of available therapies, including hormonal and nonhormonal treatments, and other are still under study. This rich scenario opens up new options that may increase the number of women for whom a suitable treatment may be available.

In the light of the new development of treatment options for VVA, we thought that it was timely to provide an updated expert opinion on the management of VVA.

## Methods

The activity comprised different steps. A panel of experts (the authors), composed by six gynecologists identified on the basis of their expertise in treating menopause-associated disorders, participation in clinical trials, and regional distribution across Italy, was assembled and held a first meeting in July 2015. First, the Expert Panel decided to perform a comprehensive literature review as a basis to produce the expert statements. In a subsequent meeting, the Expert Panel formulated a provisional set of statements. In October 2015, these statements were presented for discussion, amendment, and voting to a wider panel of 23 gynecologists with specific expertise in menopause health, selected from throughout Italy. First, the whole group voted on the level of agreement with each provisional statement using a 9-point numerical rating scale (1 = do not agree at all, 9 = agree completely). Statements reaching the average score of at least 8 were accepted straightforward, while those obtaining a mean score <8 were subjected to further discussion, amended consequently, voted again, and eventually accepted if obtaining a mean score ≥8.

## Results and discussion

Most statements obtained a high level of agreement (≥8) from the first vote. Minor changes, in terms of rewording, better specifications, and clarifications, were introduced to the few statements that did not reach immediate consensus, and at the second vote all statements reached an agreement score ≥8. The approved final statements are presented and discussed hereafter.

*1. VVA, currently framed within the GSM, includes genital and sexual symptoms involving vulva and vagina with anatomical-functional changes related to age and lack of estrogens.*

Portman et al. [6] first defined GSM as a collection of symptoms and signs associated with a decrease in estrogen and other sex steroids involving changes to external genitals, urethra, and bladder. The syndrome may include but is not limited to genital symptoms of dryness, burning, and irritation; sexual symptoms of lack of lubrication, discomfort or pain, and impaired function; and urinary symptoms of urgency, dysuria, and recurrent urinary tract infections [17]. The panel of experts thinks that the term VVA is comprised within the GSM but more specifically applies to those of the above-cited signs and symptoms that strictly involve genital organs and genito-sexual symptoms, excluding urinary organs and symptoms. From an anatomical point of view, the term VVA refers specifically to changes in the vulvovaginal surface, that on examination is thin, pale, and dry, narrowing of the vagina, especially in the absence of sexual activity, thinning of the vaginal lining that becomes less elastic and progressively smoother as rugal folds decrease [14,20].

*2. VVA prevalence varies, according to different epidemiological studies, to the parameters considered for diagnosis, and to the age of the patients, but involves on average more than 50% of postmenopausal women. VVA usually starts soon after menopause (but sometimes even before) and its prevalence progressively increases with the number of years following menopause.*

The prevalence of sexual symptoms at the menopause differs across studies; however, most of authors have been agreeing since two decades that VVA affects more than 50% of postmenopausal women [3,7,8,21,12]. VVA starts at the menopausal transition, has a chronic progressive nature throughout the menopausal status, and is not likely to resolve without intervention [21,14]. Severe VVA can result in a vaginal surface that is progressively friable, with petechiae and ulcerations. Vulvovaginal anatomical changes occur gradually but become more severe and distressing with time and can make sexual intercourses very uncomfortable or eventually impossible [16,20].

*3. VVA diagnosis is based on anamnesis and clinical examination; however, it may be useful to integrate them with objective measures obtained through validated tools, such as the Vaginal Health Index (VHI), which includes vaginal pH measure.*

As stated above, symptoms and signs of VVA may allow diagnosis [17,20]; however, some validated tools have been developed that may be helpful by adding some objective measures to the clinical examination. Bachmann et al. [22] developed the VHI in order to objectively assess female urogenital health (Table 1), to follow it on a longitudinal basis, and to share the findings with patients by decision-making about pharmacological therapy. VHI may allow an objective and accurate assessment of aging changes occurring in a patient’s urogenital tissue. Other tools have been developed more recently. For instance, the Day-to-Day Impact of Vaginal Aging (DIVA) questionnaire is a new multidimensional structured, self-administered measure designed to facilitate the evaluation of the impact of vaginal symptoms on women’s activities of daily living, emotional well-being, sexual functioning, and self-concept and body image [23].

**Table 1.** Vaginal health index (Bachmann et al. [22]).

Score	Overall elasticity*	Fluid secretion type and consistency	pH	Epithelial mucosa	Moisture
1	None	None	6.1	Petechiae noted before contact	None, mucosa inflamed
2	Poor	Scant, thin yellow	5.6–6.0	Bleeds with light contact	None, mucosa not inflamed
3	Fair	Superficial, thin white	5.1–5.5	Bleeds with scraping	Minimal
4	Good	Moderate, thin white	4.7–5.0	Not friable, thin mucosa	Moderate
5	Excellent	Normal (white flocculent)	≤4.6	Not friable, normal mucosa	Normal

*Lower score corresponds to greater urogenital atrophy.

*4. In order to diagnose VVA, gynecologists should be proactive in investigating the presence of VVA, stimulating with specific questions, and showing availability to an open interview with the patient on her genitourinary health and the impact of VVA on her health and QoL*.

Very recent surveys [7–9,11,24] suggest the need of HCPs being proactive in order to help their patients to disclose the symptoms related to VVA. During gynecological consultation, postmenopausal women are often uncomfortable to report intimate symptoms spontaneously. Furthermore, VVA is not recognized as a medical condition, and data from qualitative research have reported that women have often felt their concerns were dismissed by HCPs as a normal part of aging, without receiving any counseling about treatment options [25]. Another factor is the poor awareness by women that effective and safe treatments may be available. Also the International Vagina Dialogue Survey [26] confirmed that there is a strong need to overcome misconceptions and get reliable information on vaginal and sexual health. When postmenopausal women are seen in the clinic, it is recommended to include a gynecological pelvic examination, if suggested by symptoms and circumstances, and to stimulate an open and sensible conversation on the topic of intimacy [14,15], as also indicated by recent clinical practice guidelines [16].

It has been previously reported that HCPs tend not to take a proactive approach to sexual health management in the middle and later life age groups, mainly because of inadequate training, constraints of time, personal attitudes, and beliefs that sex is not a priority for older women [27,28]. The expert panel strongly recommends HCPs to proactively raise the subject of VVA in their medical practice in order to encourage postmenopausal women to overcome their “taboos” and openly discuss urogenital symptoms. In the end, it is up to physicians to raise the topic of vaginal health; they will appreciate that most women will express relief and respond positively. It is appropriate to include questions about vaginal symptoms, vaginal infections, recurrent urinary tract infections, dyspareunia, and previous attempts to relieve symptoms. Of course, one should be sensitive to the presence or absence of an able sexual partner and whether the patient is really distressed by her discomfort. Depending upon the specific patient, the HCP should be able to modify his/her approach to reflect the woman’s personality and culture [26,29].

*5. Treatment of VVA should preferably begin at the onset of the first symptoms and signs of atrophic changes of the vagina, which may be quite early.*

Since VVA is a chronic, age-dependent condition that may worsen without appropriate treatment, early recognition and prompt effective treatment of VVA may enhance sexual health and the QoL of women and their partners. Treatment should be started early [2], commencing therapy at the first symptoms of urogenital atrophy, since this may prevent the development of the vicious circle of worsening sexual dysfunction and urogynecological consequences [13,22].

*6. In choosing therapy, the woman’s preference should be taken into consideration.*

It must be remembered that women have individual needs, and therefore the therapeutic approach needs to be personalized. Women’s preference about treatment must be recorded and should be a major criterion in the choice of therapy [2,13,16,17]. In spite of available reassurances to the contrary, many women still fear estrogen therapy [9,11,12]. Some patients may be uncomfortable with vaginal estrogens. There are now several treatment options available, that should be fully presented and explained to the patients in order to meet their individual preferences.

*7. The use of vaginal moisturizers and lubricants and the maintenance of sexual activity may be helpful in improving vaginal dryness-related symptoms. However, a few clinical trials have been performed to assess the efficacy of such products.*

Therapies to alleviate symptoms of VVA include nonhormonal vaginal lubricants and moisturizers, as well as regular sexual activity. Regular use of nonhormonal vaginal lubricants and moisturizers may reduce friction-related irritation of atrophic tissue during vaginal intercourse, at least in breast cancer survivors with severe VVA, but they may provide only transient benefit [30]. However, women with a sexual partner should be informed that regular sexual activity/intercourse or other vaginal stimulation helps to increase the likelihood that sexual activity will remain comfortable in the future. A number of over-the-counter (OTC) vaginal lubricants and moisturizers are available. However, few clinical studies have been conducted to demonstrate their efficacy. One randomized, controlled, but short-term study demonstrated effectiveness of a pH-balanced gel compared with placebo in women treated for breast cancer. Mild irritation following administration was reported [31]. Other two studies have shown that, although vaginal moisturizers are not as effective in resolving vaginal dryness as hormonal treatments, they can significantly decrease symptoms for many women [32,33]. Another study that examined the safety of personal moisturizers and lubricants found that a number of water-based gels are hyperosmolar, inducing epithelial cellular toxicity [34]. There are very few data on the effects of lubricants that contain flavors (sugar), warming properties, or solvents and preservatives such as propylene glycol and parabens [35]. Further controlled clinical trials are required to provide adequate evidence of efficacy and safety of such products [36].

*8. Treatments with vaginal estrogens proved to be efficacious, but are associated with poor compliance (inconvenience, vaginal discharge, difficult application, smell, and loss of spontaneity during intercourse).*

For decades, vaginal estrogens have been the gold standard for treatment of symptomatic VVA. Estrogens delivered locally have been for long time the preferred mode of delivery when vaginal symptoms are the only complaint. Low-dose vaginal estrogen therapy can provide sufficient estrogen to relieve symptoms with minimal systemic absorption [16]. The results of the CLOSER survey showed that the use of local estrogen treatment for vaginal discomfort had a positive impact on relationships between postmenopausal women and their partners, common benefits of treatment including less painful and more satisfying sex for both women and their partners [11]. However, there are women who find it uncomfortable or do not want, due to personal or cultural reasons, use vaginal products. In the EU REVIVE survey [12], the most frequent disliked aspects of treatment were the route of administration (“do not like to touch body”, “not discrete”) or the messiness. The fear of hormones was common in postmenopausal women using vaginal prescription products. More than half of participants showed a clear preference for an oral pill instead of a vaginally administered product for the treatment of VVA problems. Approved in both the US and Europe the orally administered selective estrogen receptor modulator (SERM) ospemifene is a new treatment option for women. Evidence of efficacy of ospemifene is based on randomized controlled trials, and long-term safety data are available. The approved indication in Europe is for “treatment of moderate to severe symptomatic vulvar and vaginal atrophy (VVA) in postmenopausal women who are not candidates for local vaginal estrogen therapy” [17,37,38].

*9. There are not enough data on the safety of topical estrogens in patients with a history of breast cancer or of any other estrogen-sensitive tumor. Therefore, such treatments should be avoided or prescribed only following a deep discussion on benefits and risks.*

Data about low-dose topical estrogen therapy in women with breast cancer are scarce, and their use is formally contraindicated in women with (a history of) breast cancer [39]. In view of these concerns, the EMAS 2015 guidelines recommend that nonhormonal lubricants and moisturizers should be considered first-line [17]. The NAMS 2013 position paper states that for women with a history of breast or endometrial cancer, management depends on a woman’s preference, need, understanding of potential risks, and consultation with her oncologist (Level of evidence C) [16]. Thus, vaginal estrogens are controversial in women with estrogen-dependent neoplasia, and extensive discussion with the patient on the risk–benefit ratio as well as co-management with the woman’s oncologist is encouraged. The new compound ospemifene does not have this contraindication, if prescribed as directed after the completion of the treatment plan for breast cancer. Further investigation should be dedicated to the management of menopausal disturbances in women with breast cancer.

*10. The gynecologist should consider alternative therapeutic options for all those patients who are not candidate to topical estrogens.*

There will be some women who do not wish to use estrogens, or in whom estrogens are genuinely contraindicated. The development of novel products, proven to be both efficacious and safe, is therefore welcome. Palacios and Mejias [19] recently reviewed the drugs for the treatment of menopausal symptoms, reporting that there are increasing numbers of new therapies, but most of them still have inconclusive results. Vaginal laser needs more evidence regarding its efficacy and (long-term) safety. Therapies with testosterone and dehydroepiandrosterone (DHEA) are still under study [19]. Ospemifene represents currently an approved oral alternative option for symptomatic VVA.

*11. Since VVA is a chronic condition, therapy should be continued over time, because signs and symptoms recur in most cases.*

There are no guidelines pertaining to the length of therapy. For local estrogen therapy, there are no clinical trial safety data extending beyond 12 months, but no time limits for duration of therapy have been established. Since symptoms commonly return when treatment is discontinued, treatment should not be time-limited [15,18]. Appropriate monitoring should be considered. The latest NAMS position paper recommends that if a woman is at high risk of endometrial cancer or is using a higher dose of vaginal estrogens, transvaginal ultrasound or intermittent progestogen therapy may be considered. On the other hand, there are insufficient data to recommend routine annual endometrial surveillance in asymptomatic women using vaginal estrogens (Level of evidence C) [16]. The same paper states that with appropriate clinical surveillance, vaginal estrogens or ospemifene can be continued as long as bothersome symptoms are present (Level C). In their very recent update on VVA management, Palacios et al. state that in general it is probably prudent to review women’s needs annually to assess efficacy and acceptability of therapy, while updating on any new advice, information, or treatment options [18].

## Conclusions

VVA is a chronic, age-dependent condition occurring as a consequence of postmenopausal estrogen deficiency, which may worsen in the absence of appropriate treatment, leading to progressive sexual dysfunction and potentially severe urogynecological consequences. Women are often reluctant to consult or complain about VVA, so that, despite its high prevalence, this condition remains poorly recognized and undertreated by HCPs. This expert opinion has been produced with the objective of expanding and better clarifying the current recommendations, in order to help gynecologists in facing, recognizing, and promptly managing VVA in their clinical practice. In particular, we strongly recommend that HCPs proactively start an open discussion with their postmenopausal patients about urogenital symptoms. Treatment should be started as early as the first symptoms of VVA occur and should be maintained over time, due to the chronicity of the conditions. Many treatment options are now available and therapy should be individualized, taking the woman’s preference in consideration. We believe that gynecologists can do much to improve VVA by giving sufficient time and attention to this condition, thereby restoring sexual health and QoL of the many menopausal women who still suffer in silence.
